# Reducing the Breast Cancer Risk and Radiation Dose of Radiography for Scoliosis in Children: A Phantom Study

**DOI:** 10.3390/diagnostics10100753

**Published:** 2020-09-25

**Authors:** Manami Nemoto, Koichi Chida

**Affiliations:** 1 Course of Radiological Technology, Health Sciences, Tohoku University Graduate School of Medicine, 2-1 Seiryo, Aoba, Sendai 980-8575, Miyagi, Japan; manami.s@med.tohoku.ac.jp; 2 Department of Radiation Disaster Medicine, International Research Institute of Disaster Science, Tohoku University, 468-1 Aramaki Aza-Aoba, Aoba, Sendai 980-0845, Miyagi, Japan

**Keywords:** breast cancer, scoliosis, cancer risk, full-spinal radiograph, effective dose, pediatric X-ray examination, radiation safety, radiation dose, disaster medicine

## Abstract

Full-spinal radiographs (FRs) are often the first choice of imaging modality in the investigation of scoliosis. However, FRs are strongly related to breast cancer occurrence due to multiple large-field radiographic examinations taken during childhood and adolescence, which may increase the risk for breast cancer in adulthood among women with scoliosis. The purpose of this study was to consider various technical parameters to reduce the patient radiation dose of FRs for scoliosis. To evaluate breast surface doses (BSDs) in FRs, radio photoluminescence dosimeters were placed in contact with a child phantom. Using the PC-based Monte Carlo (PMC) program for calculating patient doses in medical X-ray examinations, the breast organ dose (BOD) and the effective dose were calculated by performing Monte Carlo simulations using mathematical phantom models. The BSDs in the posteroanterior (PA) view were 0.15–0.34-fold those in the anteroposterior (AP) view. The effective dose in the PA view was 0.4–0.61-fold that in the AP view. BSD measurements were almost equivalent to the BODs obtained using PMC at all exposure settings. During FRs, the PA view without an anti-scatter grid significantly reduced the breast dose compared to the AP view with an anti-scatter grid.

## 1. Introduction

The patient radiation dose and occupational exposure are significant issues in medicine [1,2,3,4,5]. Radiation doses related to medical imaging and interventional radiology procedures have been investigated [6,7,8,9,10,11]. Diagnosis and management of scoliosis require the use of conventional X-ray examinations (full-spinal radiographs (FRs)) to visualize the entire spine. FRs provide a good overview with relatively detailed morphological information, and are useful for measuring the Cobb angle, which is an indicator of the progress of spinal curvature (i.e., the progress of scoliosis) [12]. Children suffering from scoliosis are usually examined using FRs on a number of occasions, and examinations of the spine involve relatively large X-ray fields. Idiopathic scoliosis occurs more often in relatively young (10- to 15-year-old) females. After FRs for scoliosis, such patients who have experienced repeated procedures may be at increased risk for future radiation-induced breast cancer [13,14,15,16,17,18,19]. Therefore, physicians and radiological technologists should be aware of the need to reduce the radiation dose when examining scoliosis patients using FRs [14,20,21,22,23]. Generally, radiation protection using a lead shielding device is effective. However, in scoliosis patients with spinal curvature, adequately protecting the breast using lead shielding is inconvenient because shielding would also cover the spine, preventing the full spine from appearing in the X-ray image. 

Because children are more radiosensitive than adults, it is also important to measure the radiation dose and estimate the cancer risk [24,25,26,27,28].

We investigated various technical parameters (tube voltage, distance, and with/without a grid) with positioning (posteroanterior (PA) or anteroposterior (AP)) to reduce the patient radiation dose when using FRs for scoliosis. Measurements using a phantom and a dosimeter as well as dose-calculating software (PC-based Monte Carlo program for calculating patient doses in medical X-ray examinations (PMC)) based on the Monte Carlo method were used for the comparisons. We also estimated the effective dose for FRs under various conditions (technical parameters and positioning).

## 2. Materials and Methods 

### 2.1. Evaluation of BSDs during FRs 

**Figure 1:**
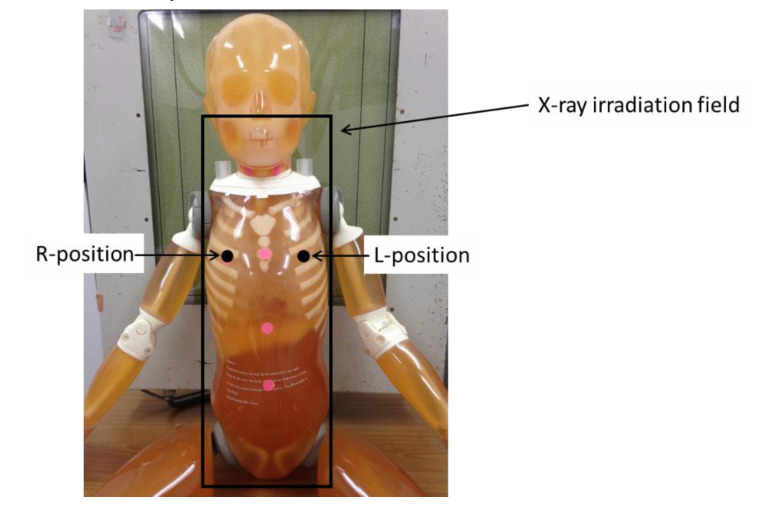
Geometric arrangement of measurements of breast surface doses (BSDs). Radio photoluminescence dosimeters (RPLDs) (GD-302M) were placed on the breast surface at the R-position and the L-position. The average values of the R-position and L-position measurements were used in this study.

A diagnostic X-ray system (DHF-155H; Hitachi, Japan) was used. Radio photoluminescence dosimeters (RPLDs) (GD-302M), provided by ChiyodaTechnol (Tokyo, Japan), were used to measure surface doses. The RPLDs were calibrated to the Japan National Standard Exposure Dose and were placed on the breast surface of a child phantom (Figure 1) so that they included backscatter radiation. The child phantom, provided by Kyoto Kagaku (Kyoto, Japan), was assumed to represent a 5-year-old patient. The phantom thickness was approximately 16.7 cm at the chest and approximately 16 cm at the abdomen. Soft tissue-equivalent material and bone-equivalent material with the same X-ray absorption as the human body were used [29].

For the RPLD measurements, the dosimeters were first annealed for 1 h at 400 °C. After X-ray irradiation, they were preheated for 40 min at 70 °C in a special oven and then measured using a reader. The radiation dose was obtained by subtracting the radiation dose in the pre-dose (i.e., background radiation) from the post-dose.

The entrance surface kerma of the image receptor was also measured using a semiconductor radiation detector (X2 System; RaySafe, Gothenberg, Sweden) to determine the X-ray conditions (output, i.e., mAs value) with and without an anti-scatter grid. The X2 detector was placed between the image receptor and the phantom, in the center of the radiation field. The mAs values required to maintain the entrance surface kerma of the image receptor (X2 measurements) were determined at approximately 25 μGy (with a grid) and 10 μGy (without a grid).

This study was performed while changing the tube voltage and focus image-receptor distance (FID) in a stepwise manner. Table 1 shows the examination configuration for projection radiographs of the FRs in this study. The irradiation field was the whole spinal column on the child phantom (Figure 1). No Pb shielding devices (lead covering) were used, and no X-ray collimation was used. Two types of beam projections were used: AP and PA. All measurements were performed three times, and the average value was used for the analyses.

### 2.2. Calculation of the Effective Doses of FRs Using the Monte Carlo Method

The effective doses (related to the factor of cancer risk) of FRs were determined with the help of commercially available PC software (PMC), developed by the Finnish Centre for Radiation and Nuclear Safety (Säteilyturvakeskus). The entrance surface air kerma of the phantom, needed for calculating the PMC, was measured using the X2 system.

Table 2 shows the technical parameters for the FRs used in the PMC calculations. Tube voltage, FID, beam projection, and irradiation field were the same as those given in Table 1. In the Monte Carlo simulations, a total of 10,000,000 individual photon histories were considered for each radiation field. In this manner, it was possible to keep the stochastic error (standard division) of organ doses to less than 1% [30].

The breast organ doses (BODs) of FRs were also determined using the PMC. We compared BODs using PMC and BSDs obtained from RPLDs (GD-302M), to determine to what degree the acquired BSDs differed from the organ doses.

## 3. Results

### 3.1. BSDs during FRs

**Figure 2:**
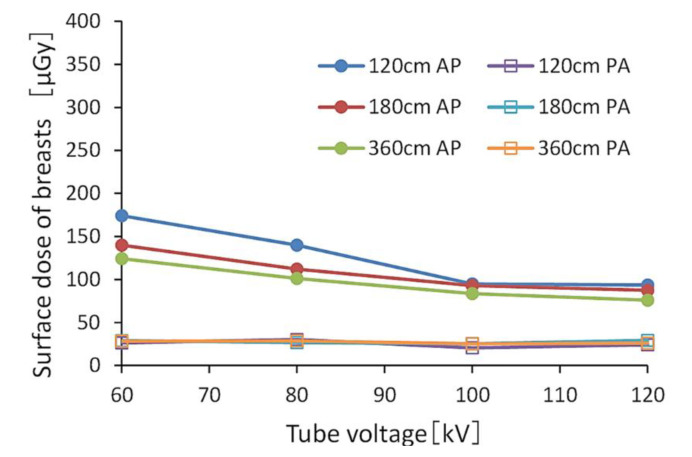
Breast surface doses (BSDs) when changing the tube voltage without a grid. (AP: anteroposterior, PA: posteroanterior).

**Figure 3:**
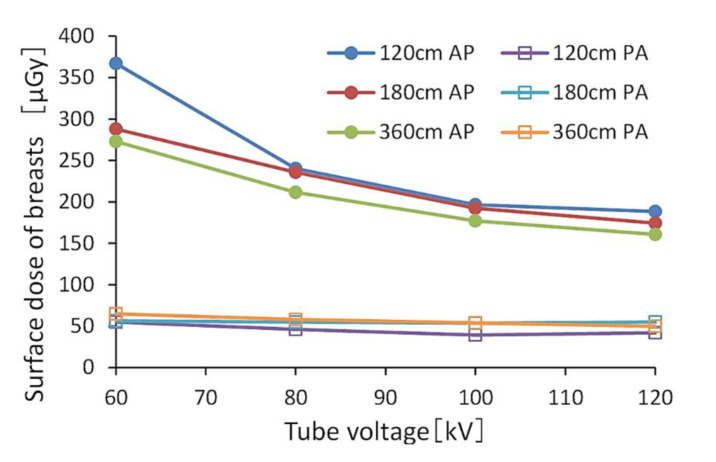
Breast surface doses (BSDs) when changing the tube voltage with a grid. (AP: anteroposterior, PA: posteroanterior).

Figure 2 and Figure 3 show the BSDs using FRs, indicating the relationships between tube voltage when the grid was or was not used. The ranges of BSDs in the AP view with and without the grid were 160.6–367.0 μGy and 75.8–174.3 μGy, respectively.

The BSD for the PA view was 0.15–0.34-fold that of the AP view. When comparing doses with and without the grid, the BSD without the grid was 0.46–0.58 times that of the grid in the AP view, and 0.51 times that in the PA view. The BSD decreased as the tube voltage and FID increased. Table 3 shows the relative values of the BSDs in the AP view when referenced (normalized) at 60 kV (tube voltage). Table 4 also shows the relative values of BSDs in the AP view when referenced (normalized) at 120 cm (FID).

### 3.2. Effective Doses during FRs Using PMC Based on the Monte Carlo Method

**Figure 4:**
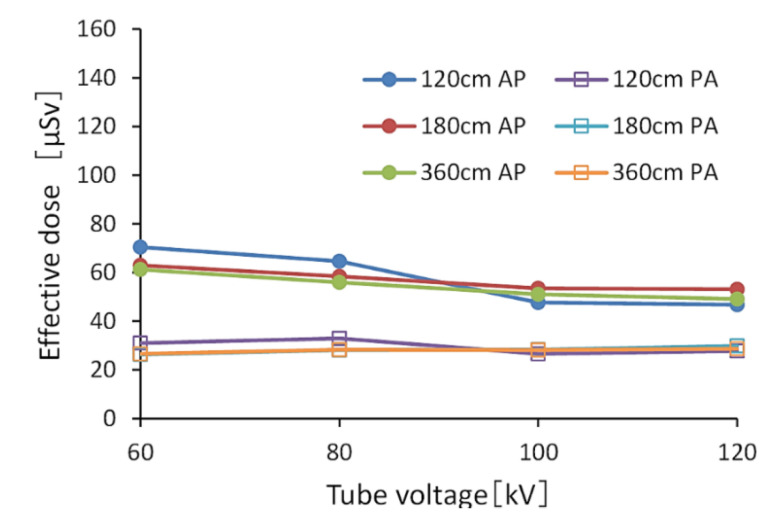
The effective dose calculated by the PC-based Monte Carlo program for calculating patient doses in medical X-ray examinations (without a grid). (AP: anteroposterior, PA: posteroanterior).

**Figure 5:**
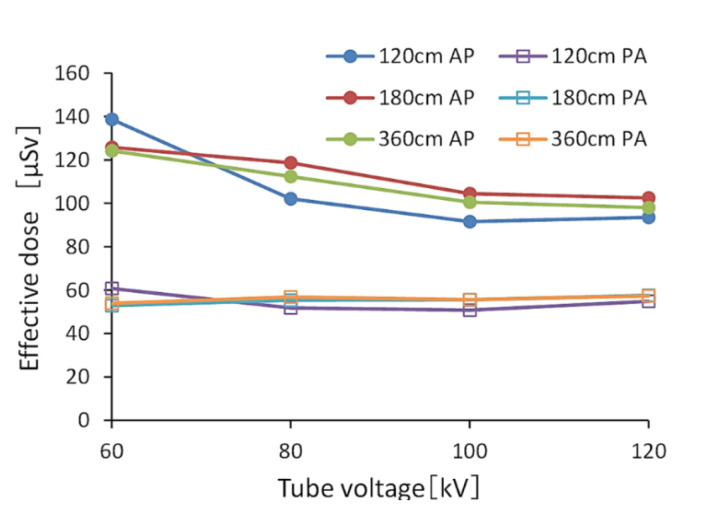
The effective dose calculated by the PC-based Monte Carlo program for calculating patient doses in medical X-ray examinations (with a grid). (AP: anteroposterior, PA: posteroanterior).

Figure 4 and Figure 5 show the effective doses calculated by PMC. Without a grid, the range of values of the effective dose in the AP view was 46.8–70.4 μSv, and the average value of an effective dose in the PA view was approximately 28.6 μSv. Using a grid, the range of values of an effective dose in the AP view was 91.7–138.8 μSv, and the average value of an effective dose in the PA view was approximately 55.3 μSv. The effective doses in the PA view were 0.4–0.61 times that in the AP view.

**Figure 6:**
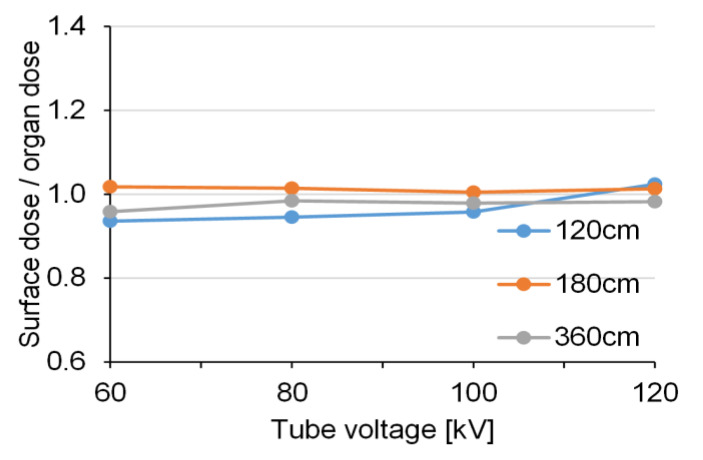
The relative values of the measured breast surface doses (BSDs) and the calculated breast organ doses (BODs) in the anteroposterior (AP) view (without a grid).

**Figure 7:**
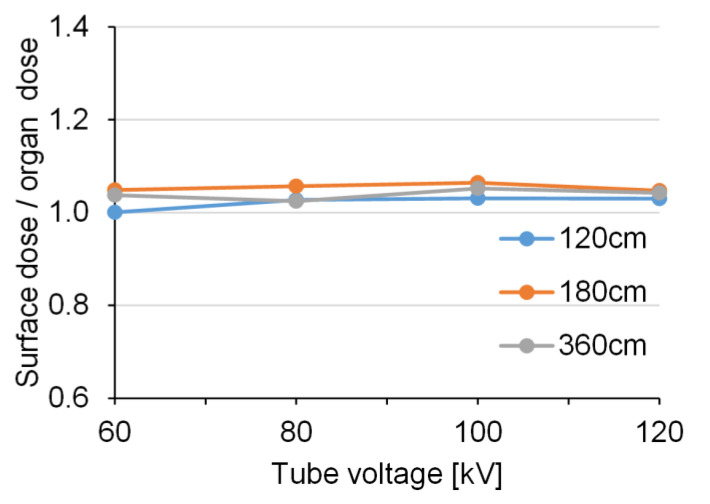
The relative values of the measured breast surface doses (BSDs) and the calculated breast organ doses (BODs) in the anteroposterior (AP) view (with a grid).

Figure 6 and Figure 7 show the relative values of the BSDs using RPLD and the calculated BODs using PMC in the AP view. The measured BSDs were approximately equivalent to the calculated BODs at all exposure settings.

**Figure 8:**
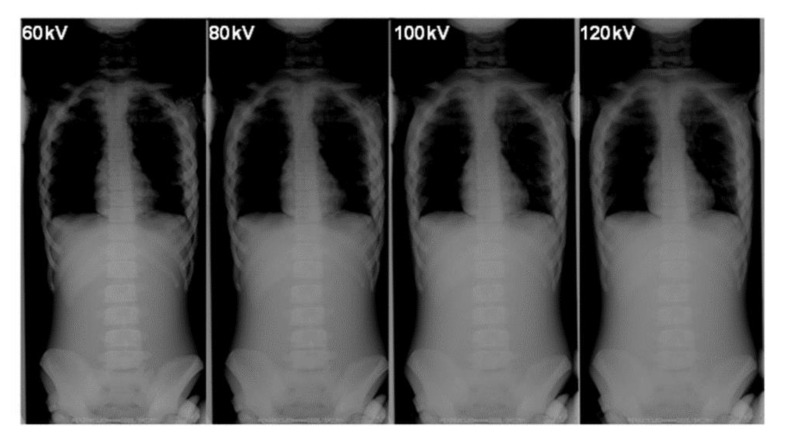
The differences in the phantom image contrast of full spinal radiographs using various tube voltages (focus-image-receptor distance: 180 cm, without a grid).

**Figure 9:**
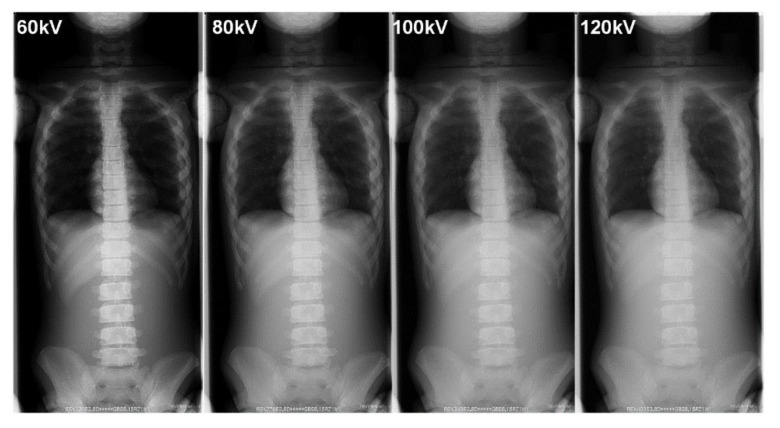
The differences in the phantom image contrast of full-spinal radiographs using various tube voltages (focus-image-receptor distance: 180 cm, with a grid).

Table 5 summarizes the BSDs and the technical parameters during the FRs. Figure 8 and Figure 9 show examples of FRs taken in our phantom research.

## 4. Discussion

We investigated patient radiation dose-reducing technical parameters with the aim of reducing the risk of breast cancer from FRs. The radiation dose was measured using a pediatric phantom and a dosimeter. We also estimated the effective dose using Monte Carlo software. First, the most effective way to minimize radiation exposure to the breast during FRs was to select imaging in the PA direction. When imaging was changed from the AP direction to the PA direction, the surface dose decreased 0.15–0.31-fold, so a dose reduction of at least 70% and a maximum of 85% was achieved. Based on the results of our study, to reduce the patient radiation dose, it is recommended to replace AP projection with a corresponding PA projection. Furthermore, to reduce BSDs during FRs, it was important to change from using the grid to not using the grid. Under the conditions in our study, not using the grid reduced the BSD by a maximum of 0.58-fold and by an average of 0.49-fold.

The effective dose is one indicator for radiation-induced cancer risk. To reduce the effective dose during FRs, it was also very important to use the PA view, without the grid.

A lower tube voltage (i.e., 60 kV) resulted in a higher radiation dose during FRs. When using a grid and a 120 cm FID, increasing the tube voltage from 60 kV to 80 kV reduced the dose by approximately 35%. Using FID, the reduction was large only when the tube voltage was 60–80 kV; when the tube voltage was 100 kV or more, the reduction was small.

The BOD calculated using the PMC was approximately equal to the surface dose measured by the RPLDs, with a ratio range of 0.94–1.06 during FRs. These approximately equal values may have been due to the undeveloped mammary glands of 5-year-old subjects.

Currently, there is no standard indicating safe or dangerous levels of radiation as regards the BSD or BOD during FRs.

Recently, the use of advanced imaging modalities, such as CT scans, in the diagnosis of scoliosis is gradually increasing [31,32]. However, in many hospitals these modalities are not used routinely because they are expensive. Thus, FRs remain the most widely used imaging modality for the investigation of scoliosis.

It is important to keep an appropriate balance between image quality (contrast) and radiation dose in X-ray images [33,34,35,36]. Changes in the tube voltage and whether the grid is used affect the image contrast. When spinal X-ray photographs are used in orthopedic studies, it is common to use a grid for the AP view to lower the tube voltage. However, all of these conditions tend to increase exposure to the breast.

Although the X-ray image contrast decreased with increasing tube voltage, the outline of the spine could still be recognized (Figure 8 and Figure 9). Even if the contour of the spine was not clear in the lower contrast image (lower dose, i.e., high voltage without a grid), the Cobb angle, which is a clinical indicator of the progress of scoliosis, could still be measured. In addition, image-processing technology, such as bone emphasis processing, is improving because of the use of digital photography. Hence, it may be possible to further reduce radiation doses during FRs and still obtain images that can be used to measure parameters, such as the Cobb angle, for clinical diagnoses, even under conditions that result in low image quality.

Recently, there has been some advanced progress that utilizes deep learning to improve image quality while further reducing radiation dose [37,38,39]. It is likely that this approach could also be used for FRs.

In summary, FR is strongly related to breast cancer occurrence due to multiple large-field diagnostic radiographic examinations during childhood and adolescence, which may increase the risk of breast cancer among women with scoliosis. In FR, the PA view markedly reduced the dose to the breast compared with the AP view. The use of long-distance radiography (e.g., a FID of 180 cm) was also found to reduce the breast dose. High tube voltage (i.e., 100 kVp) and removal of the anti-scatter grid were particularly effective methods for reducing the breast cancer risk in pediatric scoliosis radiography. The estimated effective dose for the PA view was also found to be reduced in full-spinal conventional X-ray examinations.

### Limitation

Since this initial study used only a phantom representative of a 5-year-old, further investigation is required using phantoms representative of 10- to 15-year-olds.

## 5. Conclusions

FRs deliver a significant radiation dose to children. In addition, they may increase the risk for breast cancer in adulthood among females with scoliosis, therefore, when using FRs for scoliosis, reducing the radiation dose (and cancer risk) is an important issue. We investigated the technical parameters for reducing the radiation dose with the aim of reducing the risk for breast cancer. During FRs, the PA view significantly reduced the dose to the breast compared to the AP view. A high tube voltage (i.e., 100 kV) and removal of the anti-scatter grid also reduced the risk. However, when the FID was greater than 180 cm, the effect of reducing the radiation dose was low. Although a high tube voltage and removal of the anti-scatter grid during FRs were effective for reducing the breast dose, it is important to balance the requirement for contrast with a reduction in the patient dose. In addition, measurements of BSDs using a dosimeter and calculations of the effective dose with PMC are useful for determining the radiation dose during FRs for scoliosis.

## Figures and Tables

**Table 1:** Geometry and exposure settings of the full-spinal radiographs. (* A limitation of the X-ray system used in our study is that the radiation field setting must be 47 cm when the focus-image-receptor distance is 120 cm. A radiation field setting of 55 cm is not possible if the focus-image-receptor distance is 120 cm.). AP: anteroposterior, PA: posteroanterior.

Focus-Image Receptor Distance (cm)	Tube Voltage (kV)	mAs		Beam Projection	Radiation Field Setting (cm^2^)
		Grid (−)	Grid (+)		
120	60	6.4	12.6	AP/PA	20 × 47 *
	80	2.4	4		
	100	1	1.92		
	120	0.63	1.28		
180	60	12.6	25.2	AP/PA	20 × 55
	80	5	10		
	100	2.56	5		
	120	1.6	3.2		
360	60	63	128	AP/PA	20 × 55
	80	25	50		
	100	12.8	25		
	120	8	16		

**Table 2:** The parameters used in the PC-based Monte Carlo program for calculating patient doses in medical X-ray examinations (Monte Carlo method).

**Patient’s Age (years)**	5
**height (cm)**	110
**weight (kg)**	20
**Anode Angle**	12°
**Tube Voltage**	See Table 1
**Total Filtration**	2.8 mm aluminum
**Monte Carlo Simulation**	10^7^ photon histories

**Table 3:** The relative values of breast surface doses (BSDs) in the anteroposterior view (normalized at 60 kV).

	Grid (−)				Grid (+)			
	60 kV	80 kV	100 kV	120 kV	60 kV	80 kV	100 kV	120 kV
120 cm	1.00	0.80	0.54	0.54	1.00	0.65	0.53	0.51
180 cm	1.00	0.80	0.66	0.63	1.00	0.82	0.67	0.61
360 cm	1.00	0.81	0.67	0.61	1.00	0.78	0.65	0.59

**Table 4:** The relative values of breast surface doses (BSDs) in the anteroposterior view (normalized at 120 cm).

	Grid (−)			Grid (+)		
	120 cm	180 cm	360 cm	120 cm	180 cm	360 cm
60 kV	1.00	0.80	0.71	1.00	0.78	0.74
80 kV	1.00	0.80	0.72	1.00	0.98	0.88
100 kV	1.00	0.98	0.88	1.00	0.98	0.90
120 kV	1.00	0.94	0.81	1.00	0.93	0.85

**Table 5:** Parameters for full-spinal radiographs and measured breast surface doses (BSDs). (AP: anteroposterior, PA: posteroanterior).

Technical Parameters			Surface Dose [μGy]
Beam Projection	Grid	Tube Voltage [kV]	
PA	(−) (+)	60–120	25–50
AP	(−)	100–120	75–95
		60–100	95–170
	(+)	100–120	160–200
		60–100	200–290
